# Supplementary material for: Impact of Tumor Size on Prolactinoma Characteristics and Treatment Outcomes: A Study of a Tunisian Cohort
Source: Biomedicines. 2025 May 6;13(5):1125. doi: 10.3390/biomedicines13051125 (PMC12108838; doi:10.3390/biomedicines13051125)
Supplement: Supplementary file 1 [file biomedicines-13-01125-s001.zip › biomedicines-3429336-supplementary.pdf]

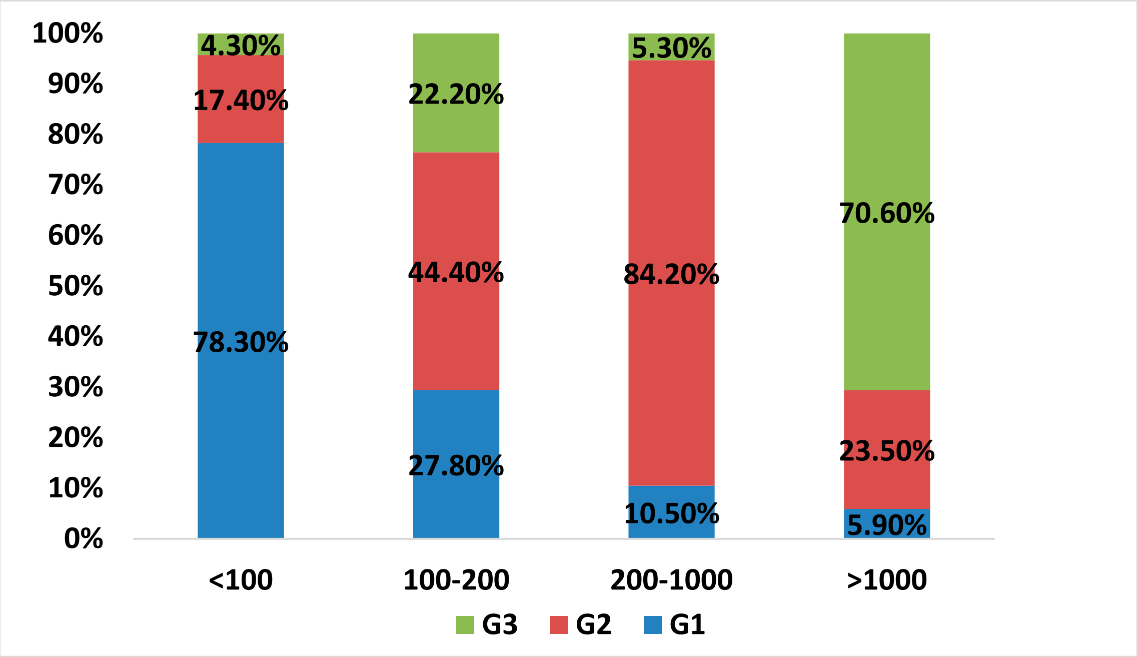

Figure S1: Distribution of prolactin level by group

Table S1: Epidemiological characteristics of study's population

Mean	Population	G1	G2	G3	p	R
**N**	77	27	32	18	0,1	-
**Sex Ratio (M/F)**	26/51	4/23	8/24	14/4	<0.001	-
**Age**	38.3 ± 14.2	31.4	41.5	42.9	0.003	0.348

Table S2: Frequency of anterior pituitary deficits

	G1	G2	G3	P
**Hypogonadotropic hypogonadism**	14(26%)	22(42.3%)	17(32.7%)	0.008
**Central hypothyroidism**	2(14.3%)	5(35.7%)	7(50%)	0.028
**Corticotroph deficiency**	3(9.4%)	14(43.8%)	15(46.9%)	0.000
**Three axes deficit**	1(9%)	4(36.3%)	6(54.5%)	0.002

Table S3: Frequency of used treatment by group

	Medical treatment only	Surgical treatment
**G1**	25(92.1%)	2(7.4%)
**G2**	20(62.5%)	12(37.5%)
**G3**	11(61.1%)	7(38.8%)
**P**	0.082	0.024

Table S4: Evolution of prolactin levels by group

	G1			G2			G3			
**Dosage timing**	N	M	%	N	M	%	N	M	%	p
**Pre - therapeutic**	26	164.1	0	32	523.4	0	18	10569.1	0	0.000
**After 1 month**	10	71.8	33.3%	11	689.7	33.3%	9	4594.9	33.3%	0.989
**After 6 months**	20	41.5	30%	23	97.8	50%	13	726.7	20%	0.598
**After 1 year**	18	56.1	50%	21	136	38.5%	5	1315	11.5%	0.283
**After 5 years**	7	11.6	43.8%	14	48.1	50%	4	104.4	6.2%	0.039
**After 10 years**	2	10.7	33.3%	5	23.7	66.7%	1	63	0%	0.155

*N : Number of assessed prolactinemia (PRL)
**M : Mean PRL (ng/ml)
***% : Percentage of normal PRL

Table S5: Distribution of remission and resistance

	Remission (N=10)	Resistance (N=10)
**Men**	4(40%)	4(40%)

**Women**	6(60%)	6(60%)
**Under cabergoline**	5(50%)	0%
**Under bromocriptine**	0%	10(100%)
**G1**	4(40%)	0%
**G2**	4(40%)	7(70%)
**G3**	2(20%)	3(30%)

Table S6: Remission parameters as a function of tumor size, dose and duration of treatment

	**Prolactin normalization during follow-up**		**>50% diameter reduction**	
	No= 32 (41.5%)	Yes=45 (58.4%)	No=42 (53.3%)	Yes=35 (46.7%)
**G1**	8(29.6%)	19(70.3%)	17(63%)	10(37.3%)
**G2**	13(40.6%)	19(59.3%)	10(31.2%)	22(68.7%)
**G3**	11(6.1%)	7(38.8%)	15(83.3%)	3(16.6%)
**P**	0,076		0.001	
**Mean maximal dose of BC (mg)**	7.72	6.71	7.07	7.4
**P**	0.39		0.49	
**Mean maximal dose of CB (mg)**	0.91	0.68	0.78	0.79
**P**	0.16		0.780	
**Total duration of treatment (months)**	37.12 (1-180)	67.91 (4-216)	31.31 (1-156)	82.85 (4-216)
**P**	0.027		<0.001	
